# Supplementary figures and images for: Pyrimidine salvage in Toxoplasma gondii as a target for new treatment
Source: Front Cell Infect Microbiol. 2023 Dec 15;13:1320160. doi: 10.3389/fcimb.2023.1320160 (PMC10755004; doi:10.3389/fcimb.2023.1320160)

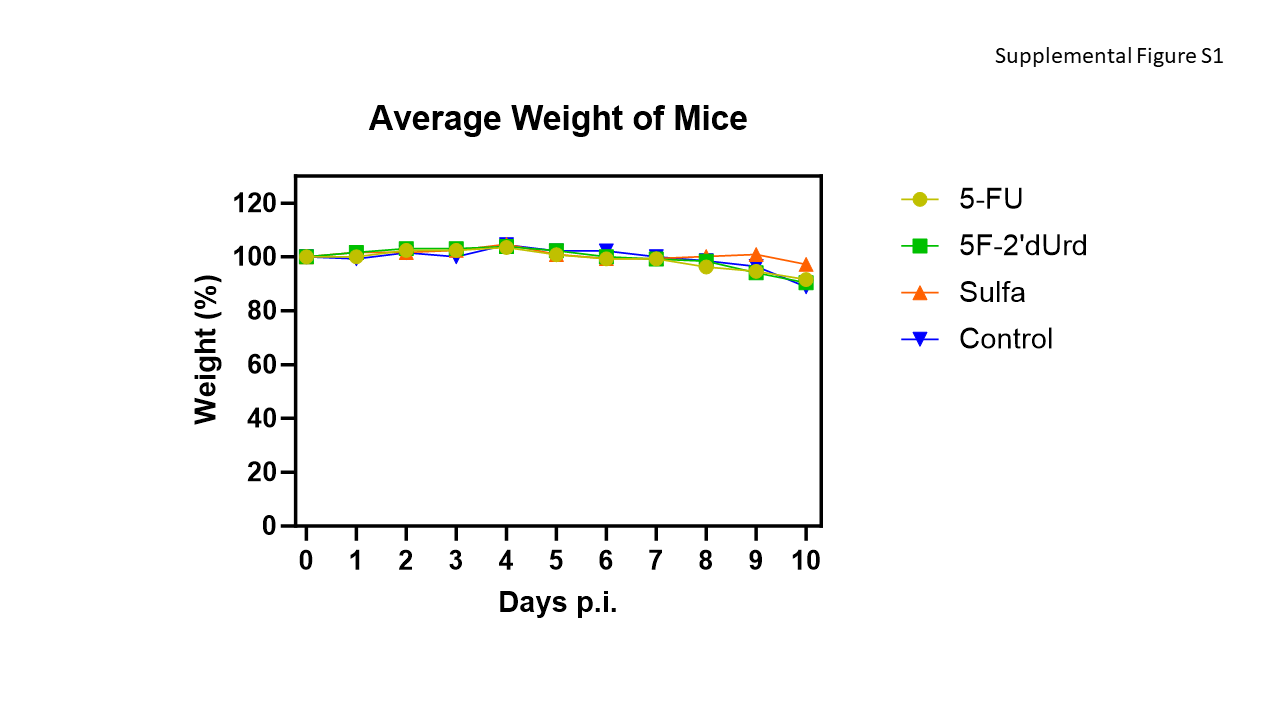

Supplement: Supplementary file 1 [file Image_1.tif]
